# Catheter Fever

**Published:** 1898-09-17

**Authors:** 


### CATHETER FEVER.

Dr. Mansell Moullin graphically describes in the *Lancet* the causes of that urinary fever which is apt to arise at the beginning of catheter life. The beginning of it all is cystitis, which is always purulent, its immediate exciting cause being invariably the access of pyogenic organisms. The symptoms are those of a subacute form of septicæmia in a person whose health is broken down by chronic renal degeneration, and they are to be prevented not by slowly and gradually reducing the amount of residual urine, but by proper attention to the principles of aseptic catheterism. It must be remembered that the fact that the urine has not undergone ammoniacal decomposition is no proof whatever that it is free from organisms, for the worst forms of septic cystitis and urinary septicæmia may occur independently of ammoniacal decomposition and with urine which is distinctly and persistently acid. The source of the organisms is the urethra. They exist there and in the neighbourhood of the meatus in profusion, and where catheterism has to be employed in cases in which there is a considerable amount of residual urine the greatest pains are necessary to prevent their being introduced into the bladder. The hands, the prepuce, and the skin of the penis should be cleansed with soap and water, as thoroughly as if a surgical operation was about to be performed, and then sponged over with a solution of corrosive sublimate, 1 to 5,000. An irrigating catheter is introduced into the fossa navicularis, which is thus washed out from behind with boric acid, the catheter being then pushed into the deeper part, and the process repeated. Finally, Melchior's double catheter is introduced, and the urine drawn off. When, however, catheters have to be used frequently, it will never be possible to ensure such precautions being carried out each time. But at least the catheters can be kept clean. The catheters employed should be as smooth and polished on the inside as on the outside, and no one catheter should be used more than once in the day. Two of the catheter cases devised by Dr. Nicoll, of Glasgow, should be made use of. In one the clean boiled catheters should be kept ready for use, and into the other, filled with boric acid solution, they should be placed directly they are removed from the urethra. Once a day all the catheters should be boiled, and transferred from the boric acid solution to the other case, in which they are to be kept clean and dry.

